# Three‐dimensional printing in radiation oncology: A systematic review of the literature

**DOI:** 10.1002/acm2.12907

**Published:** 2020-05-27

**Authors:** Michael K. Rooney, David M. Rosenberg, Steve Braunstein, Adam Cunha, Antonio L. Damato, Eric Ehler, Todd Pawlicki, James Robar, Ken Tatebe, Daniel W. Golden

**Affiliations:** ^1^ College of Medicine University of Illinois at Chicago Chicago IL USA; ^2^ Department of Radiation Oncology University of California San Francisco CA USA; ^3^ Department Medical Physics Memorial Sloan Kettering Cancer Center New York NY USA; ^4^ Department of Radiation Oncology University of Minnesota Minneapolis MN USA; ^5^ Department of Radiation Medicine and Applied Sciences University of California San Diego CA USA; ^6^ Department of Radiation Oncology Dalhousie University Halifax Canada; ^7^ Department of Physics and Atmospheric Science Dalhousie University Halifax Canada; ^8^ Radiation Medicine Program Princess Margaret Cancer Center Toronto ON Canada; ^9^ Department of Radiation and Cellular Oncology University of Chicago Chicago IL USA

**Keywords:** 3D printing, additive manufacturing, clinical application, radiation oncology

## Abstract

**Purpose/objectives:**

Three‐dimensional (3D) printing is recognized as an effective clinical and educational tool in procedurally intensive specialties. However, it has a nascent role in radiation oncology. The goal of this investigation is to clarify the extent to which 3D printing applications are currently being used in radiation oncology through a systematic review of the literature.

**Materials/methods:**

A search protocol was defined according to preferred reporting items for systematic reviews and meta‐analyses (PRISMA) guidelines. Included articles were evaluated using parameters of interest including: year and country of publication, experimental design, sample size for clinical studies, radiation oncology topic, reported outcomes, and implementation barriers or safety concerns.

**Results:**

One hundred and three publications from 2012 to 2019 met inclusion criteria. The most commonly described 3D printing applications included quality assurance phantoms (26%), brachytherapy applicators (20%), bolus (17%), preclinical animal irradiation (10%), compensators (7%), and immobilization devices (5%). Most studies were preclinical feasibility studies (63%), with few clinical investigations such as case reports or series (13%) or cohort studies (11%). The most common applications evaluated within clinical settings included brachytherapy applicators (44%) and bolus (28%). Sample sizes for clinical investigations were small (median 10, range 1–42). A minority of articles described basic or translational research (11%) and workflow or cost evaluation studies (3%). The number of articles increased over time (*P* < 0.0001). While outcomes were heterogeneous, most studies reported successful implementation of accurate and cost‐effective 3D printing methods.

**Conclusions:**

Three‐dimensional printing is rapidly growing in radiation oncology and has been implemented effectively in a diverse array of applications. Although the number of 3D printing publications has steadily risen, the majority of current reports are preclinical in nature and the few clinical studies that do exist report on small sample sizes. Further dissemination of ongoing investigations describing the clinical application of developed 3D printing technologies in larger cohorts is warranted.

## 1 INTRODUCTION

Three‐dimensional (3D) printing is an additive manufacturing technique used to generate customizable 3D objects using a variety of stock materials. Given its versatility and commercial availability, 3D printing is increasingly being incorporated into medical practice and innovation.^1^ It has been used in a diverse array of applications ranging from training and education^2^ to therapeutic medical devices.^3^ Three‐dimensional printing often represents a low‐cost alternative to traditional material fabrication methods and can also be used to generate patient‐specific models which might not otherwise be readily obtainable.^4^, ^5^ Furthermore, 3D printing allows for fabrication of certain complex geometries that is not possible by other techniques such as milling or injection molding.

Although 3D printing is recognized as an effective clinical and teaching tool in procedurally intensive medical specialties such as oral maxillofacial surgery and orthopedics,^6^ it has a nascent role in radiation oncology. Therefore, the goal of this investigation is to clarify the extent to which 3D printing applications are currently being used in radiation oncology through a systematic review of the literature. The specific aims are to comprehensively characterize current 3D printing applications in the field, identify possible areas of growth, and create a framework for encouraging safe and effective implementation of such technologies for current and future radiation oncology practitioners.

## 2 MATERIALS AND METHODS

A search protocol was defined according to preferred reporting items for systematic reviews and meta‐analyses (PRISMA) guidelines.^7^ PubMed was searched by two independent reviewers (MKR and DMR) using combinations of predetermined medical subject headings (MeSH) and generic search terms. Included MeSH terms were radiation oncology, radiotherapy, radiotherapy planning, computer‐assisted, printing, three‐dimensional, and neoplasms. Generic search terms included three‐dimensional printing, 3D printing, 3D printable, 3D printer, radiotherapy, radiation oncology, dosimetry, additive manufacturing, fused deposition modeling (FDM), and stereolithography (SLA). Fused deposition modeling and SLA are two of the popular additive manufacturing techniques performed by 3D printers. In FDM, a material is melted and extruded through a small nozzle, depositing the material layer by layer to construct the 3D‐printed object. By contrast, SLA uses a curable photopolymer material to construct objects typically in a top‐to‐bottom fashion. The material is hardened through the application of focused or UV light to generate the final product.

Search queries with less than 200 results were manually reviewed (Table S1). In‐press publications in radiation oncology and medical physics journals were individually searched (International Journal of Radiation Oncology*Biology*Physics, Radiotherapy and Oncology, Practical Radiation Oncology, Advances in Radiation Oncology, Brachytherapy, Journal of Contemporary Brachytherapy, Public Library of Science One, Journal of Applied Clinical Medical Physics, Medical Dosimetry, Medical Physics, Physics in Medicine and Biology). References of articles were searched to identify any additional studies. Searches were performed from December 2018 through February 2019 and results are reflective of available articles as of February 10, 2019.

Eligibility for inclusion of articles was determined using the population, intervention, control, outcomes (PICO) framework.^8^ The population included any peer‐reviewed publication after January 1, 2000 available in English language. This date was selected because it represents the approximate time when 3D printing was introduced and then widely adopted in medicine.^9^ Interventions included any use of 3D printing technology in relation to the study or delivery of radiotherapy. The controls, when defined, were conventional procedures as compared to 3D printing interventions, as specified in each study. Outcomes included qualitative and quantitative results described in articles, if applicable. There were no specific exclusion criteria. Any disagreements regarding inclusion of articles were discussed with the study team until consensus was reached.

Included studies were coded into a database and evaluated according to predetermined parameters of interest. End points included in this study were (a) year and country of publication, (b) description of the technology or intervention, (c) study type (randomized controlled trial, cohort study, case series or report, review or meta‐analysis, translational or basic science research, preclinical feasibility study, cost or workflow evaluation study), (d) sample size for clinical intervention studies, (e) type of radiation oncology application, (f) reported outcomes, and (g) safety concerns or implementation barriers. Studies were considered clinical intervention studies if they evaluated or tested a 3D‐printed application directly on patients. Given unknown trends in types of published 3D printing applications, the “type of radiation oncology application” end point was coded and categorized in a post hoc fashion after qualitative review of all studies. Outcomes were heterogeneous and general trends were qualitatively assessed after finalization of data collection. Coding of outcomes was performed with particular attention to (a) accuracy, (b) precision, (c) workflow efficiency, (d) cost, (e) patient experience, and (f) educational value.

Linear regression was used to analyze the rate of publication, and all statistical calculations were performed using R 3.3.2 (R Foundation, Vienna, Austria). PRISMA guidelines also recommend additional analyses such as sensitivity or subgroup analyses, as appropriate. However, such recommendations are more applicable for meta‐analyses and thus were prospectively discarded for the present investigation. Institutional Review Board approval was not required due to the nature of the study.

## 3 RESULTS

**Fig. 1:**
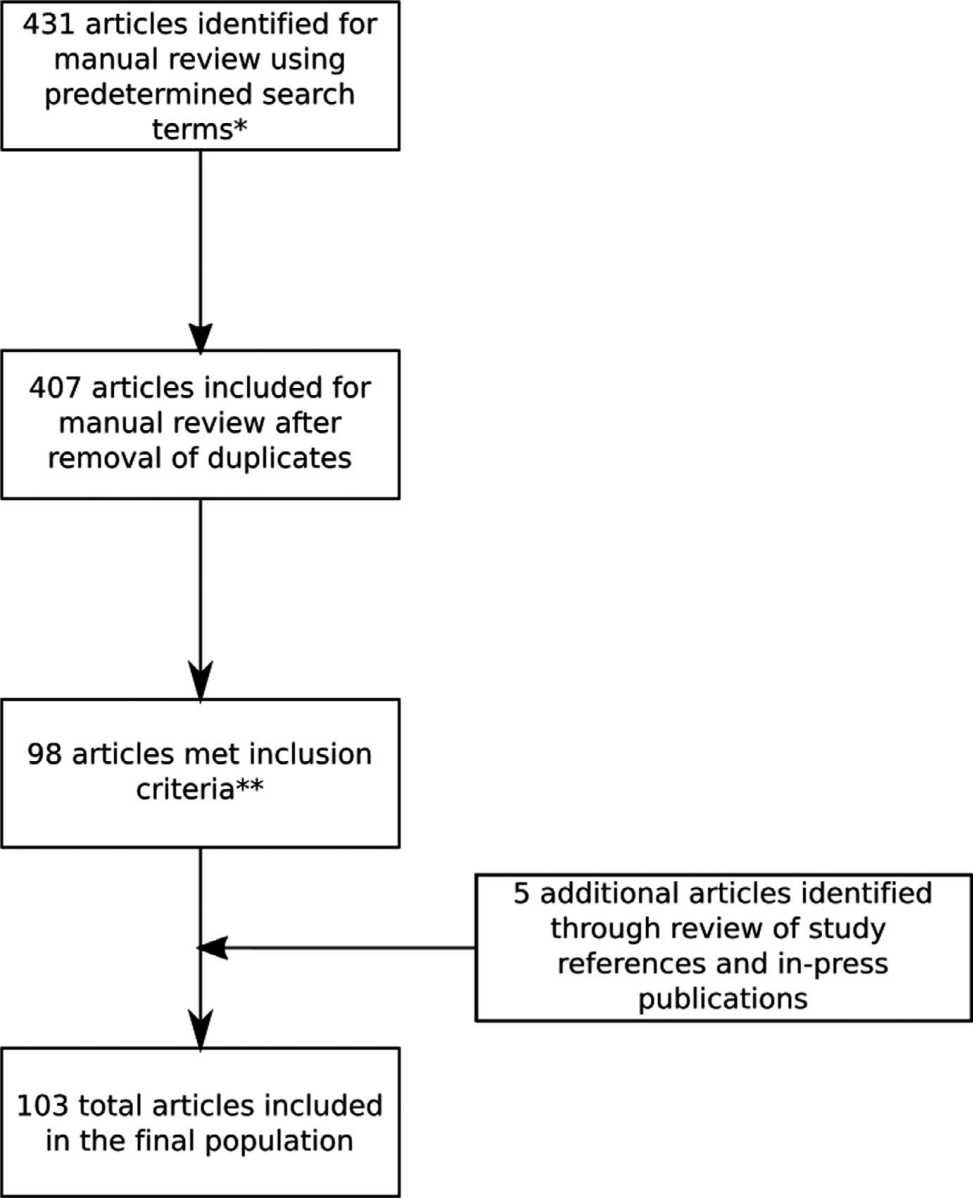
Search protocol for identification of eligible articles. *Medical Subject Headings (MeSH) terms included: radiation oncology; radiotherapy; radiotherapy planning, computer‐assisted; printing, three‐dimensional; neoplasms. Generic search terms included: three‐dimensional printing; 3D printing; 3D printable; 3D printer; radiotherapy; radiation oncology; dosimetry; additive manufacturing; fused deposition modeling (FDM); stereolithography (SLA). **Inclusion criteria were defined according to the PICO (population, intervention, control, outcomes) framework. The population included any peer‐reviewed publication after January 1, 1990 available in English. Interventions included any uses of 3D printing technology specifically in the context of radiation oncology or the delivery of radiotherapy. The control, if defined and applicable, was standard of care interventions or procedures compared to a 3D printing‐related intervention as specified in each study. Outcomes included qualitative and quantitative results described in articles, if applicable

The search protocol yielded 103 articles meeting inclusion criteria (Fig. 1). A summary of evaluation data is provided in Table 1 and a list of all identified articles is available in Table S2. The most commonly described 3D printing applications were quality assurance phantoms (26.2%), brachytherapy applicators (20.4%), bolus (16.5%), preclinical animal irradiation (9.7%), compensators (6.8%), immobilization devices (4.9%), and beam modulators (4.9%). For studies evaluating 3D‐printed bolus, 71% (12/17) used photon radiotherapy, 24% (4/17) used electron radiotherapy, and 6% (1/17) used proton radiotherapy. A minority of studies described multidisciplinary applications of 3D printing, such as evaluation of multiple interventions simultaneously (4.9%).

**Table 1:** Evaluation summary of identified articles.

Description	Count (n = 103)	%
Study type		
Preclinical feasibility study	65	63.1
Case report or case series	13	12.6
Basic science or translational research	11	10.7
Cohort Study	11	10.7
Cost or workflow evaluation	3	2.9
Radiation oncology topic		
Quality assurance phantoms	27	26.2
Brachytherapy applicators	21	20.4
Bolus	17	16.5
Photon radiotherapy	12	70.6
Electron radiotherapy	4	23.5
Proton radiotherapy	1	5.9
Preclinical animal irradiation	10	9.7
Compensator blocks	7	6.8
Immobilization	5	4.9
Beam modulators	4	3.9
Multidisciplinary	4	3.9
Other	8	7.8
Publication year		
2012	1	1.0
2013	0	0.0
2014	6	5.8
2015	14	13.6
2016	19	18.4
2017	28	27.2
2018	32	31.1
2019	3	2.9
Country of publication		
USA	26	25.2
Korea	16	15.5
Canada	13	12.6
China	8	7.8
Germany	7	6.8
Japan	6	5.8
Italy	4	3.9
UK	4	3.9
Other	19	18.4

**Fig. 2:**
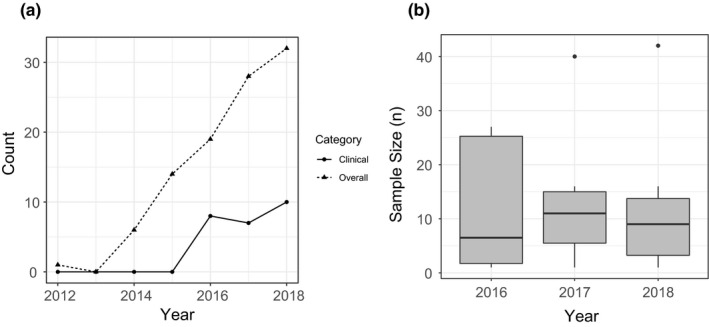
Publications describing clinical evaluations of three‐dimensional (3D) printing applications* in radiation oncology over time (a), with distributions of clinical sample sizes described individually by year (b)**. *Studies were defined as clinical if they tested or evaluated 3D‐printed applications directly on patients. Publications from 2019 were excluded because data collection were ongoing at that time. **According to the described definition above, clinical studies were only published during and after 2016; for Panel B, only this time range is displayed

Most identified publications described preclinical feasibility studies (63.1%), with fewer case report/series (12.6%), cohort studies (10.7%), basic science and translational research articles (10.7%), and cost/workflow evaluations (2.9%). While the number of publications increased by an average of 5.51 articles per year over time (*P* < 0.0001), few of these were clinical studies which were defined as the testing or evaluation of a 3D‐printed application on actual patients [24.2%, Fig. 2(a)]. Furthermore, the sample sizes for clinical studies were small, with an overall median of 10 [range 1–42, Fig. 2(b)]. A detailed description of individual clinical studies is provided in Table 2.^10–34^ The most common applications evaluated clinically included brachytherapy applicators (48%) and bolus (28%).

**Table 2:** Summary of clinical studies evaluating 3D printing applications.

Publication year	First author	Description of intervention/application	Study type	Sample size	Component of radiation oncology	Outcomes (particularly in relation to (a) accuracy, (b) precision, (c) efficiency of workflow (d) cost, (e) patient experience, (f) educational value
2016	Park^10^	Printed bolus for IMRT in a patient with Kimura's disease involving the auricle	Case report/series	1	Bolus	Observed an acceptable fit of the 3D‐printed bolus with a maximum air gap of <5 mm at the tragus. Most of the surface area of the target region was covered by the 95% isodose line. The plan with the 3D‐printed bolus improved target coverage compared to that without a bolus
2016	Canters^11^	Patient‐specific bolus for radiotherapy in the treatment of non‐melanoma skin cancer	Cohort study	26	Bolus	The V85% of the CTV was on average 97% (3D print) vs 88% (conventional). Geometric comparison of the 3D‐printed bolus to the originally contoured bolus showed high similarity (DSC = 0.89). The dose distributions on the second CT scan with the 3D print bolus in position showed only small differences in comparison to the original planning CT scan. A time benefit of the 3D print workflow was identified: one hour for the radiation oncologist, one hour for the patient and four hours for the radiation therapists
2017	Park^12^	Clinical application of printed bolus in breast electron conformal therapy	Case report/series	6	Bolus	The uniformity index on the chest skin was improved when the 3D‐printed boluses were used, with overall average skin dose being closer to the prescribed one in the former case (−0.47% vs −4.43%). 3D‐printed boluses resulted in a reduction in the mean dose to the ipsilateral lung by up to 20%
2017	Lukowiak^13^	Development and evaluation of personalized bolus used for electron therapy treatment of skin lesions of the eye canthi	Case report/series	11	Bolus	The average matching level index (0–100%, 1 SD) of the 3D‐printed boluses was 95.1 ± 2.1%, compared to 46.0 ± 10.1% for the manually fabricated paraffin bolus. Mean doses were closer to the prescribed doses, and dose spreads were less for the dose distributions from the 3D‐printed boluses
2018	Chiu^14^	Printing of silicone bolus for head and neck radiotherapy	Cohort study	7	Bolus	In vivo dose measurements on 7 treated patients confirmed that the dose deposition conformed to planned doses. Material costs were lower than currently available hard 3D‐printed plastic boluses. All treated patients tolerated the bolus for duration of treatment
2018	Ehler^15^	Clinical and workflow evaluation of 3D printed bolus using animal patients	Cost/workflow study	14	Bolus	The mean and median time from segmentation to generation of 3D‐printed boluses was 6.15 h and 5.25 h, respectively. The 3D‐printed bolus was significantly less deviated from the planned bolus compared to the conventional bolus (p = 0.0078) with measured dose under the bolus within 5% agreement of expected dose in 88% of the measurement
2018	Robar^16^	Intrapatient comparison of printed bolus versus standard vinyl gel sheet bolus for postmastectomy chest wall radiotherapy	Cohort study	16	Bolus	In 13 of 16 patients, the bolus was printed without user intervention, and the median print time was 12.6 hours. The accuracy of fit of bolus was improved, with the frequency of air gaps 5 mm or greater reduced from 30% to 13% (P < .001), and size of maximum air gaps was reduced. Surface dose was within 3% for both standard sheet and 3D‐printed bolus. Printed bolus reduced mean setup time from 104 to 76 seconds
2016	Walker^17^	Evaluation of printed sizing tools for use during intraoperative breast brachytherapy	Case report/series	2	Brachytherapy applicator sizing tool	Implementation resulted in an immediate decrease in consumable costs without affecting the quality of care or the speed of delivery
2016	Sethi^18^	Clinical evaluation of 3D printed vaginal cylinders	Case report/series	3	Brachytherapy applicators	Patient 1 received 31.5 Gy in three fractions over 2 weeks. Patient 2 received 36 Gy in six fractions over two implants on week apart. Patient 3 received 18 Gy in three fractions over one implant after external beam treatment. Brachytherapy was tolerated well with no grade 3 or higher toxicity and no local recurrence
2016	Huang^19^	Clinical evaluation of a previously developed 3D printed brachytherapy applicator for head and neck cancer	Cohort study	25	Brachytherapy applicators	Mean entrance point distance deviation for all 619 needles was 1.18 ± 0.81 mm, varying from 0.857 ± 0.545 to 1.930 ± 0.843 mm at different sites. The mean angular deviation was 2.08 ± 1.07 degrees, varying from 1.85 ± 0.93 to 2.73 ± 1.18 degrees at different sites. The addition of individual template guidance reduced the time required for implantation and minimize the damage to normal tissues
2016	Hongtao^20^	Dosimetric comparison of conventional and 3D‐printed brachytherapy applicators	Cohort study	27	Brachytherapy applicators	Mean D90 values pre‐ and post‐operation in the printed applicator group were (87.09 ± 33.63) Gy and (85.31 ± 34.40) Gy, respectively, with no statistically significant difference. The mean D90 values pre‐ and post‐operation in the traditional group were (86.04 ± 29.93) Gy and (74.96 ± 46.10) Gy, respectively, with a statistically significant difference. The mean V90 values post operation in the template and traditional groups were (92.76% ± 1.89%) and (84.59% ± 7.56%), respectively, with a statistically significant difference
2017	Liu^21^	Use of personalized 3D printed brachytherapy template for treatment of recurrent ameloblastoma of the skull base	Case report/series	1	Brachytherapy applicators	Postoperative evaluation showed that seeds were placed correctly according to the preoperative plan. On positron emission tomography 18 months later, the tumor had completely regressed
2017	Ji^22^	Dosimetric comparison of pre‐ and postoperative plans for 3D printed template assisted seed implants	Cohort study	14	Brachytherapy applicators	There was no significant difference between the two groups for all parameters (D90, minimum peripheral dose, V100, V150, and V200) except for V100. Postoperative V100 was lower than pre‐operation V100 (90.5% vs. 92.5%, p = 0.027)
2017	Ji^23^	Dosimetric comparison of pre‐ and postoperative plans for 3D printed template assisted seed implants in retroperitoneal/paravertebral tumors	Cohort study	16	Brachytherapy applicators	Compared with preplanned cases, the dose of the target volume was slightly lower, and the high‐dose area of the target volume was larger in postoperative cases without statistical significance. Actual dose conformity of the target volume was lower in postoperative plans than preplanned (p = 0.005)
2017	Han^24^	Clinical evaluation of printed brachytherapy template for liver cancer brachytherapy	Cohort study (retrospective)	40	Brachytherapy applicators	Shorter operation times and better dose distribution were observed using the printed applicator; there were no differences in treatment response between the template assisted and control groups
2018	Lancellotta^25^	Clinical report of printed mold for brachytherapy of hard palate carcinoma	Case report/series	1	Brachytherapy applicators	The mold was ready for use within 5 hours. The 3D‐printed mold provided effective dose coverage in the PTV with V95%=99.01%. HDR‐IRT treatment using the 3D mold was well tolerated with no patient discomfort reported
2018	Sekii^26^	Inversely designed printed template‐guided interstitial brachytherapy for vaginal tumors	Case report/series	2	Brachytherapy applicators	For patient 1, a mean of 10 applicators were inserted through the holes of the template in an average of 9 minutes (range, 5–15 minutes). Median D90% of the CTV was 6.34 Gy. For patient 2, all applicators were inserted through the inside of the template. The median D90% of the CTV was 7.03 Gy. No grade 3 or higher toxicity was reported
2018	Aristei^27^	Patient‐specific printed templates for high‐dose‐rate interstitial multicatheter brachytherapy in patients with breast cancer	Cohort study	13	Brachytherapy applicators	Visual assessment showed medial and lateral target volume projections, as defined by the 3D‐printed template and standard method, overlapped on the patient’s skin. Concordance was confirmed by X‐ray findings. Both methods showed the 3D‐printed target volume always fell within the standard volume in all 13 patients. The ICC was 0.48 for the medial and 0.64 for the lateral volumes, indicating moderate agreement
2018	Jiang^28^	Side effect analysis of using 3D printed template‐assisted brachytherapy in the treatment of recurrent head and neck malignancies	Cohort study	42	Brachytherapy applicators	No case had an acute reaction of grade ≥ 3. Three cases had a grade‐1 skin reaction. Blood toxicity did not occur, nor spinal cord injury. Xerostomia was not aggravated beyond that of before brachytherapy. One case had a grade‐3 nerve response. These results are comparable to established techniques
2018	Haefner^29^	Development and positional accuracy evaluation of 3D printed immobilization masks	Case report/series	8	Immobilization devices	The mean 3D displacement was 0.9 mm with a standard deviation of the systematic and random error of 0.2 mm and 0.5 mm, respectively
2018	Kuijten^30^	Printed conformer with mask to aid in the treatment of contracted sockets after radiation damage in the treatment of retinoblastoma	Case report/series	1	Mask for treatment of radiation‐related tissue contraction	As a result of the treatment, the patient can now wear a cosmetic prosthesis on average for 3 hours a day. The conjunctival lining was expanded compared to baseline. The mask and conformer were well tolerated, and no adverse effects were encountered. However, surgical intervention was still eventually required
2017	Zhao^31^	Clinical application of 3D printing for bolus fabrication and brachytherapy applicators	Case report/series	5	Multidisciplinary	Based on the planning CT, the size of the largest air gap at the interface of the 3D‐printed structure was 3 mm, 3 mm, 2 mm, and 2 mm for four cases using printed bolus. The surface brachytherapy plan for the adequate coverage (95% isodose to 95.6% of CTV]), but a relatively high dose to the left eye, owing to its proximity to the tumor
2016	Briggs^32^	Personalized radiotherapy facial protective masks using a facial scanner.	Case report/series	1	Protective shielding	The lead mask fitted comfortably and was confirmed by the lead clinician to be safe to use for treatment. The final print time was 30 hours and a total of 200 g of print material was used at an approximate cost of £8.00
2018	Sharma^33^	Clinical evaluation of lead face shielding created using an optical scanner and 3D printer	Case report/series	10	Protective shielding	Lead shields created using this approach were accurate and well‐fitting. The process added to patient convenience and addressed potential claustrophobia and medical inability to lie supine
2016	Conti^34^	Creation of patient specific models for AVM lesions as tools to aid in radiosurgery treatment planning	Cohort study	10	Radiosurgical treatment planning	Contouring time was shorter when using 3D‐printed model of the AVM than without (p = 0.001). The average volume contoured without the 3D model was 5.6 ± 3 mL whereas it was 5.2 ± 2.9 mL with the 3D‐printed model (p = 0.003). Surgeons were absolutely confident or very confident in all cases that the volume contoured using the 3D‐printed model was plausible and corresponded to the real boundaries of the lesion

Abbreviations: 3D = Three‐dimensional, AVM = arteriovenous malformation, CT = computed tomography, CTV = clinical target volume, D90 = dose received by 90 of the target organ or volume, DSC = dice similarity coefficient, Gy = Gray, HDR‐IRT = high‐dose‐rate interventional brachytherapy, ICC = intraclass correlation coefficient, PTV = planning target volume, SD = standard deviation, V85/90/100/150/200 = volume of target receiving 85/90/100/150/200 of the prescribed dose.

Reported outcomes are summarized in Table 3. Although end points were heterogeneous, the majority of studies described effective implementation of 3D printing technology. The most commonly reported outcome was dosimetric evaluation of printed interventions (50.5%). Other common end points were related to the 3D printing process itself, such as printing accuracy, time, and cost. Furthermore, many studies evaluated the radiological properties (12.6%) and durability or deformation (2.9%) of printed materials. Studies describing the use of 3D printing for preclinical animal irradiation (9.7%), such as animal immobilizers, often described animal positional accuracy (3.9%), or histologic confirmation of targeted radiotherapy delivery (2.9%). Clinical or disease‐related outcomes such as treatment toxicity (3.9%) and disease control or overall survival (1.9%) were rarely reported, reflecting the preclinical nature of many of the identified investigations.

**Table 3:** Summary of reported outcomes for included studies.

Reported outcome	Count* (n = 103)	%
Dosimetric evaluation	52	50.5
Printing or patient positional accuracy	20	19.4
Radiodensity of printed materials or simulated tissue	13	12.6
Cost/time of printing or materials	11	10.7
Workflow description or efficiency	10	9.7
Accuracy of bolus fit	9	8.7
Patient reported comfort	6	5.8
Accuracy of brachytherapy seed or catheter placement	5	4.9
Treatment toxicity	4	3.9
Animal positional accuracy	4	3.9
Histologic confirmation of accurate radiotherapy delivery (preclinical animal irradiation)	3	2.9
Material durability or deformation	3	2.9
Perceived utility by providers	2	1.9
Disease‐related clinical outcome	2	1.9
Learner comfort with brachytherapy	1	1.0
Contouring time	1	1.0

The majority of articles described at least one safety concern or implementation barrier for the use of 3D printing applications in radiation oncology (69.9%, Table 4). The most commonly described barriers were related to the 3D printing process, including limitations in time/workflow (12.6%), printing accuracy (10.7%), cost (8.7%), limited print volume (6.8%), and a requirement for printing space (1%). Additionally, 3D printing filaments (materials that are directly printed) were routinely cited sources for safety concerns and implementation barriers. Variable radiological properties (8.7%), limited biocompatibility and sterilization capacity (7.8%), dosimetric inconsistency or inaccuracy (3.9%), hardness impacting patient comfort or tissue simulation (2.9%), durability or stability (2.9%), and limited color (1.9%), flexibility (1.9%), and number of simultaneously printed filaments (1.9%) were the most commonly described concerns with current 3D printing filaments or materials. Additionally, the need for clinical validation prior to routine use (6.8%) and limited generalizability of 3D‐printed interventions for individual patient characteristics (5.8%) were commonly described barriers. One article described that there is a significant learning curve for the 3D printing fabrication process which acts as a barrier to routine clinical implementation.

**Table 4:** Implementation barriers and safety concerns when using 3D printing technology in radiation oncology.

Implementation barrier or safety concern	Count (n = 103)	%
At least one barrier or safety concern described	72	69.9
3D printing process		
Time/workflow	13	12.6
Accuracy	11	10.7
Cost	9	8.7
Limited print volume	7	6.8
Printing space	1	1.0
3D printing materials		
Variable or inaccurate radiodensity	9	8.7
Biocompatibility and sterility	8	7.8
Dosimetric variability	4	3.9
Hardness (for patient comfort and/or tissue simulation)	3	2.9
Stability/durability	3	2.9
Limited color availability	2	1.9
Limited range of flexibility	2	1.9
Limited number of materials printed at a time	2	1.9
Requires clinical validation before implementation	7	6.8
Clinical and anatomic variation across patients limits applicability of printed interventions	6	5.8
Learning curve for new users	1	1.0

## 4 DISCUSSION

To our knowledge, this investigation represents the most comprehensive systematic review describing 3D printing applications in radiation oncology. Through formal characterization of currently reported 3D printing interventions and identification of common barriers to safe and effective implementation, this study will serve as a guide for practitioners and researchers considering introducing 3D printing technology in their routine practice. Many exciting opportunities exist for the implementation of 3D printing in the radiation oncology clinic or laboratory and the majority of identified interventions appeared to successfully improve treatment delivery according to dosimetric analysis or small‐sample clinical evaluation. However, further work will be needed to confirm the efficacy of such interventions in larger clinical settings.

The most commonly described applications were quality assurance phantoms, brachytherapy applicators, and bolus (Table 1). This trend likely reflects the ability of 3D printers to fabricate relatively low‐cost patient‐specific models, which serves as one of the central advantages of 3D printing compared to traditional material fabrication methods such as casting or molding.^35^ For quality assurance phantoms, brachytherapy applicators, and bolus, the use of individualized models can improve treatment delivery and minimize unnecessary toxicity.^11^, ^24^, ^36^ For example, 3D‐printed bolus can conform more closely to patient skin compared to traditional bolus and thereby minimize inaccuracies in treatment delivery.^12^, ^13^

Such patient‐specific models are not easily created with traditional techniques or may have high cost of production and thus may not be readily available for routine use. With 3D printing technology, patient imaging data can be used to generate virtual models of individual anatomy, which can then be used as a source file for the 3D printer. A variety of imaging modalities can be used to generate patient models such as computed tomography (CT), magnetic resonance imaging (MRI), or even a 3D surface scanner from a mobile device.^33^ The resultant low‐cost, patient‐specific interventions have been shown to improve fidelity of anatomic simulation and can increase accuracy of treatment delivery as measured with dosimetric indices.^20^, ^25^ Less commonly described applications included compensator blocks, immobilization devices, and beam modulators.

Although unifying comparisons of 3D‐printed and traditional interventions are difficult given the diverse nature of currently reported applications in the literature, it is important to highlight that 3D printing techniques appear to be not only as accurate and efficient but also cost‐effective. For bolus applications, 3D printing was reported to improve conformity, dosimetric measurement, or clinical outcome compared to commercial bolus in 11 of 17 studies, while 3 reported equivalent outcomes, and 3 did not make direct comparisons. These results suggest that 3D‐printed bolus is likely equivalent, if not superior to commercial bolus; however, it is unclear whether these differences necessarily translate into meaningful clinical benefits. The evidence is less compelling in other settings, where direct comparisons were less often reported. For example, in studies of 3D‐printed brachytherapy applicators, only 4 of 21 reported improved outcomes via workflow efficiency or dosimetric evaluation of 3D‐printed applicators, while 7 of 21 described equivalent outcomes, and 10 did not have direct comparisons to conventional controls. Nonetheless, for the majority of all identified articles, the authors described at least theoretical advantages to 3D printing techniques for each particular application, such as improving patient comfort, workflow, or cost‐effectiveness. The lack of compelling comparison data reflects the immature nature of this field and highlights an important necessity for future investigation.

3D printing in radiation oncology has steadily grown in recent years (Fig. 2) and has been effectively implemented in a diverse array of applications. However, this review suggests that despite this rapid growth, there is still a significant need for clinical evaluation of 3D printing methods prior to widespread adoption. Only 24% of identified articles described the evaluation of printed interventions on actual patients and sample sizes of such studies were generally small (Table 2; Fig. 2). Furthermore, studies rarely reported objective clinical outcomes such as disease control or survival (Table 3). This pattern might be related to a lack of awareness of 3D printing applications among radiation oncology clinicians. Promotion of these techniques in the professional community would encourage further investigation of printed applications in the clinical setting, ideally with larger cohorts and increased focus on disease‐ and outcome‐related end points.

This investigation also identifies important gaps in current 3D printing research in radiation oncology and should help guide future academic efforts in the field. For example, 3D printing has gained prominence as an educational tool in a number of procedurally intensive medical specialties.^2^ However, this review identified only one study in radiation oncology which used 3D printing for educational purposes, specifically for the development of a cervical cancer brachytherapy training simulator.^37^ In surgical and procedural settings, 3D printing is used to generate models on which learners can practice complex surgical or manual skills.^3^, ^5^, ^38^ This approach offers a number of advantages over traditional teaching methods, including minimizing potential harm to patients and increasing overall training exposure, particularly in rarely encountered clinical situations.^39^, ^40^, ^41^, ^42^ Given the proven efficacy of 3D printing in other areas of medical education, this discrepancy represents an important opportunity for future innovation.

Although not typically considered a procedurally‐intensive specialty, the field of radiation oncology could benefit from incorporation of 3D printing into training. For instance, 3D‐printed patient‐specific models have been used as tools to help visualize complex arteriovenous malformations (ATMs) during radiosurgical treatment planning.^34^ Expanding on this concept, 3D printing patient‐specific organs or tumors might also help trainees visualize complex anatomical relationships when learning how to contour.

Furthermore, although not formally considered a component of graduate medical education, 3D printing could also be used as a tool to enhance patient education in radiation oncology. As an example, prior to surgical consultations, 3D‐printed models of patient anatomy and pathology have been used as tools to guide conversations and educate patients.^43^, ^44^, ^45^ Such interventions could similarly be used to help patients understand important details of radiotherapy, such as describing the use of radiosurgery for the treatment of brain metastases.

Another significant gap identified in this investigation is a lack of literature describing effective methods to implement 3D printing techniques in the laboratory and clinic. While many studies acknowledged the need for 3D printing expertise for consistent fabrication of accurate interventions, few reports explicitly described methods to teach current providers how to effectively use, design, and evaluate 3D printing applications.^46^, ^47^ Additionally, there were few studies describing the workflow systems for efficiently incorporating 3D printing into the clinic or laboratory.^15^, ^48^, ^49^ Expansion of such work would be a pivotal step in encouraging widespread dissemination of this technology.

Although this investigation highlights exciting opportunities for the application of various 3D printing interventions in radiation oncology, it is important to carefully consider common safety concerns and implementation barriers prior to employment of such technologies (Table 4). The most frequently described of such barriers involved various aspects of the 3D printing process which can require significant experience before consistently accurate performance can be achieved.^50^ Additionally, the financial and time burden can be large, including not only the cost of printers and materials themselves, but also the need for a physical printing space and training of staff. Encouragingly, the cost of printers has consistently decreased over recent years and many highly accurate printers are now available at reasonable prices.^51^ Printing times and maximum print volumes can also be restrictive, as the majority of commercially available printers can take up to 12 or more hours for a single print and may only be able to fabricate a cubic foot of material in one session. For some radiation oncology applications, these printing times and volumes may be acceptable, but for others such as full thoracic phantoms, such issues may be significantly limiting.

Similarly, although advances have been made to improve available printing filaments, there are still important disadvantages for many widely used materials. For functions such as brachytherapy applicators, in which the printed intervention comes in direct contact with patient skin, it is critical that printed materials are not only biocompatible, but also can be safely sterilized.^17^, ^52^, ^53^ For many commonly used filaments, the sterilization process can impact structure, stability, or dosimetric properties and thus may compromise clinical efficacy. Ongoing research will further characterize the impact of such processes on material properties, thereby improving patient safety and treatment delivery. Other commonly reported but less clinically significant limitations of printed materials include hardness which can cause patients discomfort^53^, ^54^ (e.g., in the setting of bolus), and limited availability of colors and translucency,^55^ which would be beneficial in contexts such as brachytherapy applicators. Again, researchers are currently developing methods to overcome such material limitations, such as the use of flexible printing filaments which would improve patient comfort when using 3D‐printed bolus. Lastly, safety assessment of 3D printing may be useful to guide future applications and standardization of best practices.^56^, ^57^

Despite the use of a structured protocol according to PRISMA guidelines, this study has methodological limitations. First, it is possible that some publications were not identified using the predetermined search terms or some articles were not included despite meeting inclusion criteria. However, the use of multiple independent reviewers minimizes the risk of significant deviations from the defined protocol. Additionally, some articles that would have otherwise met inclusion criteria but are not indexed on PubMed or available in‐press in the stated journals may not have been identified. Such articles could have potentially been identified through other database searches but for the sake of maintaining reproducibility this was not attempted. Second, an important consideration in any literature review is the risk of publication bias. For example, it is possible that investigations of 3D printing applications are more likely to be published if they report successful implementation of the technology. This might result in an overly positive impression of the efficacy of such interventions. Third, some end points included in the data collection were subjective in nature and therefore may not be entirely reproducible across reviewers. For example, described implementation barriers for the use of 3D printing may not be explicitly stated in each article and, therefore, one reviewer might interpret results differently than another. Fourth, because there was a large set of identified literature with diverse reported outcomes, it is difficult to draw overarching comparisons between 3D‐printed and conventional approaches. For example, some articles evaluated interventions through dosimetric measurement while others described positional accuracy. These are related although certainly distinct end points which makes it difficult to compare overall efficacy of these interventions in a unified manner. Nonetheless, attempts were made to identify key fundamental similarities and differences between 3D printing and traditional approaches. Lastly, for the sake of simplicity, safety concerns and implementation barriers were coded as a single end point; however, in reality these represent two distinct although related entities. For example, while biocompatibility of materials is both a safety concern and a barrier to implementation, long printing time would be more accurately categorized as an implementation barrier alone. This distinction should be considered when interpreting aggregate data reported in this study.

## 5 CONCLUSION

This systematic review comprehensively characterizes current uses of 3D printing in radiation oncology and identifies common barriers to effective implementation of the technology. 3D printing techniques have created exciting opportunities for radiation oncology innovation and have been increasingly utilized across a wide range of applications in the field. There are, however, limited data evaluating the clinical safety and efficacy of such interventions and further investigation is needed to facilitate routine adoption on a large scale. Therefore, this study provides a resource for radiotherapy practitioners considering introducing 3D printing in their practice and could guide further research efforts to expand the role of 3D printing in radiation oncology.

## CONFLICTS OF INTEREST

Dr. Golden reports having a financial interest in RadOnc Questions, LLC.

## Supporting information


**Table S1.** Search query results using combinations of predetermined search terms*
**Table S2.** List of all identified articles describing 3D printing applications in radiation oncology
