# Supplementary material for: Three‐dimensional printing in radiation oncology: A systematic review of the literature
Source: J Appl Clin Med Phys. 2020 May 27;21(8):15–26. doi: 10.1002/acm2.12907 (PMC7484837; doi:10.1002/acm2.12907)
Supplement: Supplementary file 1 — Table S1. Search query results using combinations of predetermined search terms* Table S2. List of all identified articles describing 3D printing applications in radiation oncology [file ACM2-21-15-s001.docx]

**Supplementary Table 1. Search query results using combinations of predetermined search terms***

| **Search Terms** | **Number of Results** |
| --- | --- |
| Neoplasms[Mesh] | 1946880 |
| radiotherapy | 222741 |
| Radiotherapy [mesh] | 114372 |
| dosimetry | 110372 |
| radiation oncology | 105124 |
| (("Radiotherapy"[Mesh]) OR "Radiation Oncology"[Mesh]) | 95118 |
| Neoplasms[MeSH] and Radiotherapy[MeSH] | 92294 |
| Radiotherapy Planning, Computer-Assisted[Mesh] | 17401 |
| 3D printing | 5875 |
| (additive manufacturing OR Fused Deposition modeling OR FDM OR Stereolithography OR SLA OR Customized applicator OR Customized mold OR Customized bolus) | 5454 |
| Radiation Oncology[Mesh] | 3180 |
| Printing, Three-Dimensional[Mesh] | 2773 |
| Printing, Three-Dimensional[Mesh] AND "Neoplasms"[Mesh] | 198** |
| (radiation oncology OR radiotherapy) AND (additive manufacturing OR Fused Deposition modeling OR FDM OR Stereolithography OR SLA OR Customized applicator OR Customized mold OR Customized bolus) | 120** |
| (3d printing OR Three dimensional printing) AND (Radiotherapy or Radiation Oncology) | 117** |
| 3d printing radiotherapy | 98** |
| radiotherapy AND 3d print | 90** |
| radiotherapy [Mesh] AND (additive manufacturing OR Fused Deposition modeling OR FDM OR Stereolithography OR SLA OR Customized applicator OR Customized mold OR Customized bolus) | 87** |
| radiation oncology AND 3d print | 72** |
| (("Radiotherapy"[Mesh]) OR "Radiation Oncology"[Mesh]) AND (3d printing OR 3d printer OR 3d Printable OR three dimensional printing) | 67** |
| Printing, Three-Dimensional[Mesh] AND radiotherapy | 60** |
| Printing, Three-Dimensional[Mesh] AND radiation oncology | 44** |
| Printing, Three-Dimensional[Mesh] AND dosimetry | 36** |
| fused deposition modeling AND radiation oncology | 4** |
| stereolithography AND radiation oncology | 3** |
| radiation oncology [Mesh] AND (additive manufacturing OR Fused Deposition modeling OR FDM OR Stereolithography OR SLA OR Customized applicator OR Customized mold OR Customized bolus) | 2** |
| ("Printing, Three-Dimensional"[Mesh]) AND "Radiation Oncology"[Mesh] | 1** |
| Radiation Oncology[Mesh] AND (3d printing OR 3d print OR three dimensional printing) | 1** |

***** Included Medical Subject Headings (MeSH) terms were radiation oncology, radiotherapy, radiotherapy planning, computer-assisted, printing, three-dimensional, and neoplasms. Generic search terms included three dimensional printing, 3D printing, 3D printable, 3D printer, radiotherapy, radiation oncology, dosimetry, additive manufacturing, fused deposition modeling (FDM), and stereolithography (SLA)

**Only queries with less than 200 results were manually reviewed for inclusion

**Supplementary Table 2. List of all identified articles describing 3D printing applications in radiation oncology**

| **First author** | **Title** |
| --- | --- |
| Arenas | Individualized 3D scanning and printing for non-melanoma skin cancer brachytherapy: a financial study for its integration into clinical workflow |
| Arimura | A feasibility study of a hybrid breast-immobilization system for early breast cancer in proton beam therapy |
| Aristei | Individualized 3D-printed templates for high-dose-rate interstitial multicathether brachytherapy in patients with breast cancer |
| Avelino | Use of 3D-printers to create intensity-modulated radiotherapy compensator blocks |
| Bache | Investigating the accuracy of microstereotactic-body-radiotherapy utilizing anatomically accurate 3D printed rodent-morphic dosimeters |
| Briggs | 3D printed facial laser scans for the production of localised radiotherapy treatment masks - A case study |
| Burleson | Use of 3D printers to create a patient-specific 3D bolus for external beam therapy |
| Canters | Clinical implementation of 3D printing in the construction of patient specific bolus for electron beam radiotherapy for non-melanoma skin cancer |
| Cervino | A novel 3D-printed phantom insert for 4D PET/CT imaging and simultaneous integrated boost radiotherapy |
| Chiu | MR-CBCT image-guided system for radiotherapy of orthotopic rat prostate tumors |
| Chiu | Three-dimensional printer-aided casting of soft, custom silicone boluses (SCSBs) for head and neck radiation therapy |
| Conti | 3D-Printing of Arteriovenous Malformations for Radiosurgical Treatment: Pushing Anatomy Understanding to Real Boundaries |
| Craft | Material matters: Analysis of density uncertainty in 3D printing and its consequences for radiation oncology |
| Craft | Preparation and fabrication of a full-scale, sagittal-sliced, 3D-printed, patient-specific radiotherapy phantom |
| Cunha | Evaluation of PC-ISO for customized, 3D Printed, gynecologic 192-Ir HDR brachytherapy applicators |
| Dancewicz | Radiological properties of 3D printed materials in kilovoltage and megavoltage photon beams |
| Diamantopoulos | Theoretical and experimental determination of scaling factors in electron dosimetry for 3D-printed polylactic acid |
| Diamantopoulos | Three dimensional printed electron beam modifier for total skin electron treatments |
| Dipasquale | Improving 3D-printing of megavoltage X-rays radiotherapy bolus with surface-scanner |
| Ehler | Workload implications for clinic workflow with implementation of three-dimensional printed customized bolus for radiation therapy: A pilot study |
| Ehler | Patient specific 3D printed phantom for IMRT quality assurance |
| Elter | Compatibility of 3D printing materials and printing techniques with PAGAT gel dosimetry |
| Epel | Spin Lattice Relaxation EPR pO2 Images May Direct the Location of Radiation Tumor Boosts to Enhance Tumor Cure |
| Epel | Oxygen-guided radiation therapy |
| Esplen | Technical note: Manufacturing of a realistic mouse phantom for dosimetry of radiobiology experiments |
| Fujimoto | Efficacy of patient-specific bolus created using three-dimensional printing technique in photon radiotherapy |
| Furdova | Early experiences of planning stereotactic radiosurgery using 3D printed models of eyes with uveal melanomas |
| Gallas | An anthropomorphic multimodality (CT/MRI) head phantom prototype for end-to-end tests in ion radiotherapy |
| Gear | Abdo-Man: a 3D-printed anthropomorphic phantom for validating quantitative SIRT |
| Haefner | 3D-Printed masks as a new approach for immobilization in radiotherapy - a study of positioning accuracy. |
| Han | Therapeutic value of 3-D printing template-assisted <sup>125</sup>I-seed&nbsp;implantation in the treatment of malignant liver tumors |
| Harris | A feasibility study for using ABS plastic and a low-cost 3D printer for patient-specific brachytherapy mould design. |
| Hazelaar | Using 3D printing techniques to create an anthropomorphic thorax phantom for medical imaging purposes |
| Homolka | Design of a head phantom produced on a 3D rapid prototyping printer and comparison with a RANDO and 3M lucite head phantom in eye dosimetry applications. |
| Hongtao | Dosimetry study of three-dimensional print template-guided precision (125)I seed implantation. |
| Hrycushko | Technical Note: System for evaluating local hypothermia as a radioprotector of the rectum in a small animal model |
| Huang | Accuracy evaluation of a 3D-printed individual template for needle guidance in head and neck brachytherapy. |
| Ji | Dosimetry verification of radioactive seed implantation for malignant tumors assisted by 3D printing individual templates and CT guidance. |
| Ji | Dosimetry Verification of (125)I Seeds Implantation With Three-Dimensional Printing Noncoplanar Templates and CT Guidance for Paravertebral/Retroperitoneal Malignant Tumors. |
| Jiang | Side effects of CT-guided implantation of (125)I seeds for recurrent malignant tumors of the head and neck assisted by 3D printing non co-planar template. |
| Jones | Introduction of novel 3D-printed superficial applicators for high-dose-rate skin brachytherapy. |
| Ju | New technique for developing a proton range compensator with use of a 3-dimensional printer |
| Jung | Verification of Accuracy of CyberKnife Tumor-tracking Radiation Therapy Using Patient-specific Lung Phantoms |
| Kadoya | Evaluation of deformable image registration between external beam radiotherapy and HDR brachytherapy for cervical cancer with a 3D-printed deformable pelvis phantom |
| Kamomae | Three-dimensional printer-generated patient-specific phantom for artificial in vivo dosimetry in radiotherapy quality assurance |
| Kim | Characterization of 3D printing techniques: Toward patient specific quality assurance spine-shaped phantom for stereotactic body radiation therapy |
| Kim | A customized bolus produced using a 3-dimensional printer for radiotherapy |
| Kim | Establishing a process of irradiating small animal brain using a CyberKnife and a microCT scanner |
| Kim | A patient-specific three-dimensional couplant pad for ultrasound image-guided radiation therapy: a feasibility study |
| Kong | A dosimetric study on the use of 3D-printed customized boluses in photon therapy: A hydrogel and silica gel study |
| Kuijten | Three-dimensionally Printed Facial Mask to Aid the Treatment of Contracted Socket After Radiation Damage in Retinoblastoma |
| Lancellotta | Individual 3-dimensional printed mold for treating hard palate carcinoma with brachytherapy: A clinical report |
| Lee | A depth-sensing technique on 3D-printed compensator for total body irradiation patient measurement and treatment planning |
| Lee | Comparing phase‐ and amplitude‐gated volumetric modulated arc therapy for stereotactic body radiation therapy using 3D printed lung phantom |
| Leong | Technical Note: The design, construction, and evaluation of a liquid-based single phantom solution for TG128 brachytherapy ultrasound QA |
| Lindegaard | Individualised 3D printed vaginal template for MRI guided brachytherapy in locally advanced cervical cancer |
| Lindsay | Design and application of 3D-printed stepless beam modulators in proton therapy |
| Lindsay | 3D printed plastics for beam modulation in proton therapy |
| Liu | I(125) brachytherapy guided by individual three-dimensional printed plates for recurrent ameloblastoma of the skull base |
| Lukowiak | Utilization of a 3D printer to fabricate boluses used for electron therapy of skin lesions of the eye canthi |
| Madamesila | Characterizing 3D printing in the fabrication of variable density phantoms for quality assurance of radiotherapy |
| Mayer | 3D printer generated thorax phantom with mobile tumor for radiation dosimetry |
| McCarroll | 3D-Printed Small-Animal Immobilizer for Use in Preclinical Radiotherapy |
| Meyer | A framework for clinical commissioning of 3D-printed patient support or immobilization devices in photon radiotherapy |
| Michiels | Patient-specific bolus for range shifter air gap reduction in intensity-modulated proton therapy of head-and-neck cancer studied with Monte Carlo based plan optimization |
| Michiels | Towards 3D printed multifunctional immobilization for proton therapy: Initial materials characterization |
| Michiels | Production of patient-specific electron beam aperture cut-outs using a low-cost, multi-purpose 3D printer |
| Nattagh | A training phantom for ultrasound-guided needle insertion and suturing |
| Niebuhr | Technical Note: Radiological properties of tissue surrogates used in a multimodality deformable pelvic phantom for MR-guided radiotherapy |
| Niebuhr | The ADAM-pelvis phantom—an anthropomorphic, deformable and multimodal phantom for MRgRT |
| Oh | Development of patient-specific phantoms for verification of stereotactic body radiation therapy planning in patients with metallic screw fixation |
| Otani | High-dose rate intracavitary brachytherapy pretreatment dwell position verification using a transparent applicator |
| Pallotta | ADAM: A breathing phantom for lung SBRT quality assurance |
| Park | Development and dosimetric assessment of a patient-specific elastic skin applicator for high-dose-rate brachytherapy |
| Park | Three-dimensional customized bolus for intensity-modulated radiotherapy in a patient with Kimura's disease involving the auricle |
| Park | Total body irradiation with a compensator fabricated using a 3D optical scanner and a 3D printer |
| Park | Clinical application of 3D-printed-step-bolus in post-total-mastectomy electron conformal therapy |
| Park | Fabrication of malleable three-dimensional-printed customized bolus using three-dimensional scanner |
| Park | A Patient-Specific Polylactic Acid Bolus Made by a 3D Printer for Breast Cancer Radiation Therapy |
| Perks | Anthropomorphic Phantoms for Confirmation of Linear Accelerator-Based Small Animal Irradiation |
| Pham | Radiotherapy Immobilization Mask Molding Through the Use of 3D-Printed Head Models |
| Poulin | Towards real-time 3D ultrasound planning and personalized 3D printing for breast HDR brachytherapy treatment |
| Ricotti | 3D-printed applicators for high dose rate brachytherapy: Dosimetric assessment at different infill percentage |
| Ricotti | Dosimetric characterization of 3D printed bolus at different infill percentage for external photon beam radiotherapy |
| Ringbæk | Validation of new 2D ripple filters in proton treatments of spherical geometries and non-small cell lung carcinoma cases |
| Robar | Intrapatient study comparing 3D printed bolus versus standard vinyl gel sheet bolus for postmastectomy chest wall radiation therapy |
| Robinson | Organ-specific SPECT activity calibration using 3D printed phantoms for molecular radiotherapy dosimetry |
| Sekii | Inversely designed, 3D-printed personalized template-guided interstitial brachytherapy for vaginal tumors |
| Sethi | Clinical applications of custom-made vaginal cylinders constructed using three-dimensional printing technology |
| Sharma | Low-cost optical scanner and 3-dimensional printing technology to create lead shielding for radiation therapy of facial skin cancer: First clinical case series |
| Su | Design and production of 3D printed bolus for electron radiation therapy |
| Tran-Gia | Design and Fabrication of Kidney Phantoms for Internal Radiation Dosimetry Using 3D Printing Technology |
| Walker | Manufacture and evaluation of 3-dimensional printed sizing tools for use during intraoperative breast brachytherapy |
| Wilke | Design and fabrication of a 3D-printed oral stent for head and neck radiotherapy from routine diagnostic imaging |
| Woods | Quality assurance for a six degrees-of-freedom table using a 3D printed phantom |
| Wu | Technical note: A 3D-printed phantom for routine accuracy check of Gamma Knife Icon HDMM system |
| Yea | Feasibility of a 3D-printed anthropomorphic patient-specific head phantom for patient-specific quality assurance of intensity-modulated radiotherapy |
| Yoon | A precision 3D conformal treatment technique in rats: Application to whole-brain radiotherapy with hippocampal avoidance |
| Zarghami | Technical Note: Immunohistochemical evaluation of mouse brain irradiation targeting accuracy with 3D-printed immobilization device |
| Zavan | Verification of Acuros XB dose algorithm using 3D printed low-density phantoms for clinical photon beams |
| Zhao | Clinical applications of 3-dimensional printing in radiation therapy |
| Zhu | Technical Note: Fabricating Cerrobend grids with 3D printing for spatially modulated radiation therapy: A feasibility study |
| Zou | Potential of 3D printing technologies for fabrication of electron bolus and proton compensators |
